# Transcriptomic Analysis Identifies *CDKN2A*-Associated Transcriptional Alterations in Primary Melanocytes of Lueyang Black-Boned Chicken

**DOI:** 10.3390/ani16162483

**Published:** 2026-08-10

**Authors:** Fan Li, Zhi Cao, Qimeng Zhang, Wei Han, Longbing Yang, Jiuzhou Song, Qian Xue, Kai Jin

**Affiliations:** 1 Joint International Research Laboratory of Agriculture and Agri-Product Safety of Ministry of Education of China, Yangzhou University, Yangzhou 225009, China; mz120241679@stu.yzu.edu.cn (F.L.); dx120240182@stu.yzu.edu.cn (Z.C.); mz120251670@stu.yzu.edu.cn (Q.Z.); 2 Jiangsu Institute of Poultry Science, Yangzhou 225125, China; hanwei830@163.com; 3 Key Laboratory for Evaluation and Utilization of Livestock and Poultry Resources (Poultry), Ministry of Agriculture and Rural Affairs, Yangzhou 225125, China; 4 Department of Animal & Avian Sciences, University of Maryland, College Park, MD 20742, USA; lyang414@umd.edu (L.Y.); songj88@umd.edu (J.S.); 5 Key Laboratory of Animal Breeding Reproduction and Molecular Design for Jiangsu Province, College of Animal Science and Technology, Yangzhou University, Yangzhou 225009, China; 6 College of Bioscience and Biotechnology, Yangzhou University, Yangzhou 225009, China

**Keywords:** chicken, primary melanocytes, *CDKN2A*, melanogenesis, gene regulation

## Abstract

Pronounced melanin enrichment characterizes the Lueyang black-boned chicken, yet the transcriptional basis underlying this pigmentation phenotype remains incompletely understood. We established a standardized in vitro culture system for primary avian melanocytes to investigate *CDKN2A*-associated transcriptional changes related to melanocyte cellular states and pigmentation-related gene expression. Primary melanocytes were isolated and characterized by morphology, ferrous sulfate staining, and TRP2 immunofluorescence, with passages P2–P4 showing relatively stable cellular characteristics. Using *CDKN2A* overexpression and knockdown models combined with RNA-seq analysis, we identified transcriptional alterations associated with *CDKN2A* modulation. Notably, *CDKN2A* overexpression was associated with increased expression of cell cycle-related genes (e.g., *CDKN1A*) and melanogenesis-associated transcriptional markers, particularly *MITF*, suggesting an association between *CDKN2A* expression and pigment-related transcriptional programs. Conversely, *CDKN2A* knockdown produced opposite expression patterns. Gene set enrichment analysis (GSEA) further indicated that high *CDKN2A* expression was associated with enrichment of melanogenesis and MAPK signalling pathways, whereas *CDKN2A* knockdown was associated with enrichment of Wnt signalling pathways. By minimizing potential in vivo paracrine effects, this in vitro model suggested that *CDKN2A*-associated transcriptional changes might reflect alterations related to cell cycle status and melanocyte cellular states. Although this study provided transcript-level evidence, direct functional assays of melanin production were not performed; therefore, these findings should be interpreted as correlative rather than definitive evidence of *CDKN2A*-associated pigmentation regulation. These findings provide a transcriptomic resource for investigating the relationship between cell-state regulation and pigmentation-associated programs in avian melanocytes.

## Simple Summary

The Lueyang black-boned chicken is a unique Chinese poultry breed known for its distinctive dark skin and meat, which are valued for cultural, nutritional, and economic reasons, as well as for breed conservation. However, the biological mechanisms controlling this dark pigmentation are not fully understood. In this study, we established a reliable laboratory system to grow and study pigment-producing skin cells from chickens and used it to investigate the role of a gene called *CDKN2A*, which is traditionally known for regulating cell growth and cellular state regulation. Our results showed that altering *CDKN2A* expression was associated with changes in the expression of a key pigment-related transcription factor and genes involved in cellular state regulation processes. These findings suggest that *CDKN2A* expression may be associated with cellular state regulation and pigmentation-related transcriptional programs in chicken melanocytes. Overall, this study provides a useful experimental model for studying pigmentation in poultry and offers new insights into how genetic factors influence coloration traits. These results may support future breeding and conservation efforts for this important chicken breed and contribute to a better understanding of how genes control visible traits in animals.

## 1. Introduction

Feather and skin pigmentation in poultry serves as an important phenotypic marker for breed identification, evolutionary biology, and studies of genetic diversity. It also significantly influences consumer preference and the economic value of poultry products. As a unique indigenous breed in China, the Lueyang black-boned chicken is characterized by pronounced melanin deposition across the skin and multiple tissues, making it a representative model for studying hyperpigmentation traits in avian species [1,2]. Previous studies in black-boned chickens have shown that melanin not only defines their characteristic phenotype but is also associated with meat quality, antioxidant capacity, and various biological functions [3,4].

In poultry, melanin deposition exhibits remarkable spatiotemporal specificity. In Lueyang black-boned chickens, feather pigmentation gradually accumulates from distal to proximal regions during development, whereas skin pigmentation initially appears in the dorsal region and subsequently extends toward the limbs and ventral side, often displaying localized aggregation patterns. Such spatial heterogeneity suggests that complex regulatory processes involving melanocyte migration, proliferation, and cellular state regulation may contribute to pigment formation. Therefore, the Lueyang black-boned chicken provides a valuable model for further understanding the biological mechanisms underlying vertebrate pigmentation [5,6].

Melanocytes are the primary functional cells responsible for melanin synthesis and deposition. Thus, establishing a stable and reliable in vitro culture system is essential for mechanistic studies. However, compared with mammalian systems, primary melanocyte culture in avian species remains technically challenging. During isolation, fibroblasts and keratinocytes may be co-isolated, potentially affecting the cellular composition of cultures. In addition, melanocytes tend to undergo morphological changes and functional decline associated with cellular senescence under in vitro conditions, which limits long-term experimental stability [7]. Therefore, the establishment of a reliable primary melanocyte culture system with stable short-term characteristics is a critical prerequisite for elucidating the molecular regulatory mechanisms underlying avian pigmentation. Melanogenesis is closely associated with the cellular state of melanocytes, including changes in proliferation, maturation, and senescence-associated processes. During melanocyte development, the balance between cellular maintenance and activation of pigmentation-related programs is accompanied by coordinated changes in gene expression profiles [8]. Therefore, genes involved in cell cycle regulation may represent potential factors linking melanocyte cellular states with pigmentation-associated transcriptional programs.

Among cell cycle regulatory factors, the cyclin-dependent kinase inhibitor *CDKN2A* has attracted increasing attention. *CDKN2A* is a well-known regulator of the G1/S cell-cycle transition and plays an important role in controlling cellular proliferation [9]. In various biological systems, proliferation and cellular state transitions are maintained in a dynamic equilibrium. During pigmentation processes, melanocyte populations must maintain appropriate cellular states while activating pigmentation-related transcriptional programs. Emerging evidence suggests that *CDKN2A* expression is associated with cellular signalling networks, including MAPK and Wnt/β–catenin pathways [10,11,12]; however, whether these pathways participate in *CDKN2A*-associated pigmentation-related responses remains unclear.

Based on these considerations, the present study aimed to establish a reliable primary melanocyte culture system from Lueyang black-boned chickens. Therefore, *CDKN2A* overexpression and knockdown models were constructed in vitro, followed by transcriptomic sequencing and expression analyses of cell cycle-related and melanogenesis-related genes, including *MITF*, *TYR*, *TYRP1*, and *KIT*, as well as related pathway-associated gene sets. This study provided transcriptional evidence indicating that *CDKN2A* may be associated with pigmentation-related and cell-state-related transcriptional changes in avian melanocytes and proposed *CDKN2A* as a candidate factor for further investigation in poultry pigmentation biology.

## 2. Materials and Methods

### 2.1. Vector Construction

Chicken *CDKN2A* (Gene ID: 395077) knockdown and overexpression vectors were constructed for *CDKN2A* expression modulation experiments. For *CDKN2A* knockdown, three shRNA sequences targeting different regions of the chicken *CDKN2A* coding sequence were designed and cloned into the PGMLV-HU6-MCS-CMV-ZsGreen1-PGK-Puro vector (Applied Biological Materials Inc., Zhenjiang, China). Recombinant plasmids were verified by Sanger sequencing, and the shRNA construct exhibiting the highest knockdown efficiency was selected for subsequent experiments.

For *CDKN2A* overexpression, the full-length coding sequence of chicken *CDKN2A* was amplified based on the NCBI reference sequence. The stop codon was removed to allow in-frame fusion with the downstream mCherry reporter, and the amplified sequence was cloned into the PGMLV-CMV-MCS-mCherry-PGK-Puro vector (Applied Biological Materials Inc., Zhenjiang, China). The recombinant plasmid was confirmed by Sanger sequencing.

### 2.2. DF-1 Cell Transfection

DF-1 chicken fibroblast cells were obtained from the cell repository of Yangzhou University and maintained in complete medium containing high-glucose Dulbecco’s modified Eagle’s medium (DMEM; Thermo Fisher Scientific, Waltham, MA, USA; Cat. No. C11995500BT) supplemented with 10% fetal bovine serum (Thermo Fisher Scientific, Waltham, MA, USA; Cat. No. SH30406.05) and 1% penicillin–streptomycin solution (Solarbio, Beijing, China; Cat. No. P1400-100 mL). Cells were cultured at 37 °C in a humidified atmosphere containing 5% CO_2_. When cells reached approximately 60–70% confluence, plasmid transfection was performed using Lipofectamine™ 3000 reagent (Thermo Fisher Scientific, Carlsbad, CA, USA; Cat. No. L3000015) according to the manufacturer’s instructions. Briefly, plasmid DNA was mixed with Lipofectamine™ 3000 reagent in Opti-MEM medium (Thermo Fisher Scientific, Waltham, MA, USA; Cat. No. 31985062) to generate transfection complexes, which were subsequently added to DF-1 cells. At 24–48 h after transfection, vector expression was evaluated based on ZsGreen1 or mCherry fluorescence signals using fluorescence microscopy (Olympus Corporation, Tokyo, Japan; Model No. TH4-200). DF-1 cells were used only for preliminary validation of vector expression efficiency and were not used for functional evaluation of melanogenesis-associated phenotypes.

### 2.3. Isolation and Culture of Primary Melanocytes

Primary melanocytes were isolated from Lueyang black-boned chicken embryos at embryonic day 12.5. All animal procedures were approved by the Institutional Animal Care and Use Committee of Yangzhou University (Approval No. 202102064). After feather removal, dorsal skin and peritoneal tissues were collected under sterile conditions and minced into small fragments. The tissues were enzymatically dissociated using 0.2% neutral collagenase type II (Solarbio, Beijing, China; Cat. No. C8151) and 0.25% trypsin–EDTA (Gibco, Thermo Fisher Scientific, Waltham, MA, USA; Cat. No. 25200072) at 37 °C. The resulting cell suspension was filtered through a 70 μm cell strainer and cultured in Human Epidermal Melanocyte Complete Medium (Pocell, Wuhan, China; Cat. No. CM-H108) at 37 °C in a humidified atmosphere containing 5% CO_2_. Primary melanocytes were characterized by cellular morphology, ferrous sulfate staining, and TRP2 immunofluorescence staining (Olympus Corporation, Tokyo, Japan; Model No. FV1200). DF-1 chicken fibroblast cells were used for preliminary evaluation of vector expression, whereas subsequent pigmentation-associated analyses were performed using primary melanocytes.

### 2.4. Ferrous Sulphate Staining

Primary melanocytes isolated from Lueyang black-boned chicken embryos were characterized using a commercial melanin staining kit based on the ferrous sulfate method (Solarbio, Beijing, China; Cat. No. G2031) according to the manufacturer’s instructions. Cells exhibiting intracellular dark brown/black melanin deposits were considered melanin-positive cells. Staining results were observed and photographed using an optical microscope (Olympus Corporation, Tokyo, Japan; Model No. TH4-200).

### 2.5. Indirect Immunofluorescence Staining

Indirect immunofluorescence staining was performed to detect TRP2 expression and cellular localization in primary melanocytes. Cells were fixed, permeabilized, and blocked before incubation with anti-TRP2 primary antibody (Proteintech, Wuhan, China; Cat. No. 13095-1-AP). After washing, cells were incubated with Alexa Fluor^®^ 488-conjugated secondary antibody (Abcam, Cambridge, UK; Cat. No. ab150077). Nuclei were counterstained with DAPI. Fluorescence images were captured using a fluorescence microscope (Leica Microsystems CMS GmbH, Wetzlar, Germany; Model No. DMi8).

### 2.6. Transfection of Primary Melanocytes

Primary melanocytes were transfected with *CDKN2A* knockdown or overexpression plasmids using Lipofectamine™ 3000 reagent (Thermo Fisher Scientific, Carlsbad, CA, USA; Cat. No. L3000015) according to the manufacturer’s instructions. Transfection efficiency was monitored by fluorescence microscopy (Olympus Corporation, Tokyo, Japan; Model No. TH4-200), and cells were subsequently collected for RNA extraction and molecular analyses [13].

### 2.7. Total RNA Extraction

Total RNA was extracted from *CDKN2A* knockdown or overexpression primary melanocytes using TRIzol™ reagent (Thermo Fisher Scientific, Carlsbad, CA, USA; Cat. No. 15596026) according to the manufacturer’s instructions. RNA concentration and purity were assessed using a NanoDrop spectrophotometer (Thermo Fisher Scientific, Wilmington, DE, USA; Model No. ND-1000). DNase treatment was performed to remove genomic DNA contamination before reverse transcription and RNA-seq library preparation [14].

### 2.8. RT-qPCR Analysis

Quantitative real-time PCR (qRT-PCR) was performed to determine the mRNA expression levels of *CDKN2A* and melanogenesis-associated genes, including *MITF*, *TYR*, *TYRP1*, and *KIT*. First-strand cDNA was synthesized from total RNA using TransScript^®^ All-in-One First-Strand cDNA Synthesis SuperMix for qPCR (TransGen Biotech, Beijing, China; Cat. No. AT341-02) according to the manufacturer’s instructions. qRT-PCR was performed using TransStart^®^ Tip Green qPCR SuperMix (TransGen Biotech, Beijing, China; Cat. No. AQ601-02) according to the manufacturer’s instructions. Relative gene expression levels were calculated using the 2^−ΔΔCT^ method, with primer sequences listed in Table 1.

### 2.9. Transcriptome Sequencing and Analysis

Total RNA was extracted from primary melanocytes, and three independent biological replicates were prepared for each experimental group. RNA quality was assessed using a NanoDrop spectrophotometer (Thermo Fisher Scientific, Waltham, MA, USA) and an Agilent 2100 Bioanalyzer (Agilent Technologies, Santa Clara, CA, USA). Samples with an RNA integrity number (RIN) ≥ 7.0 and sufficient RNA concentration were used for library construction. RNA libraries were prepared using the NEBNext Ultra RNA Library Prep Kit for Illumina (NEB, Ipswich, MA, USA) according to the manufacturer’s protocol and sequenced on the Illumina NovaSeq 6000 platform (Illumina, San Diego, CA, USA) with paired-end 150 bp (PE150) sequencing.

Raw sequencing reads were processed using fastp (version 0.23.2) to remove adapter sequences and low-quality reads. Sequencing quality was assessed using FastQC (version 0.11.9). Clean reads were aligned to the chicken reference genome *Gallus gallus* GRCg7b using STAR (version 2.7.10a). Gene expression counts were generated using RSEM (version 1.3.1) and normalized using the median-of-ratios method implemented in DESeq2.

Differential expression analysis was performed using DESeq2. Genes with an adjusted *p*-value < 0.05 and |log_2_FoldChange| ≥ 1 were considered differentially expressed. Multiple testing correction was performed using the Benjamini–Hochberg method. Functional enrichment analyses, including GO, KEGG, and GSEA, were conducted to identify biological processes and pathway-associated gene sets related to *CDKN2A* expression changes. The transcriptomic results were interpreted as *CDKN2A*-associated transcriptional alterations rather than direct regulatory effects.

### 2.10. Data Processing and Statistical Analysis

All cellular and molecular biology experiments were performed using at least three independent biological replicates. Fluorescence microscopy images were processed using ImageJ (version 1.53, National Institutes of Health, Bethesda, MD, USA) software. Statistical analyses and graphical presentations were performed using GraphPad Prism (version 9.0, GraphPad Software, San Diego, CA, USA). Transcriptomic data processing and visualization were performed using R software (version 4.2.1, R Foundation for Statistical Computing, Vienna, Austria), utilizing packages such as ggplot2 and tidyverse.

Experimental data are presented as mean ± standard deviation (SD). Data distribution and variance homogeneity were evaluated before applying parametric tests. Comparisons between two groups were performed using an unpaired Student’s *t*-test, whereas comparisons among multiple groups were performed using one-way analysis of variance (ANOVA) followed by Tukey’s multiple-comparison test when appropriate. A *p*-value < 0.05 was considered statistically significant.

## 3. Results

### 3.1. Construction of CDKN2A Expression Intervention Vectors and Their Validation in DF-1 Cells

**Figure 1:**
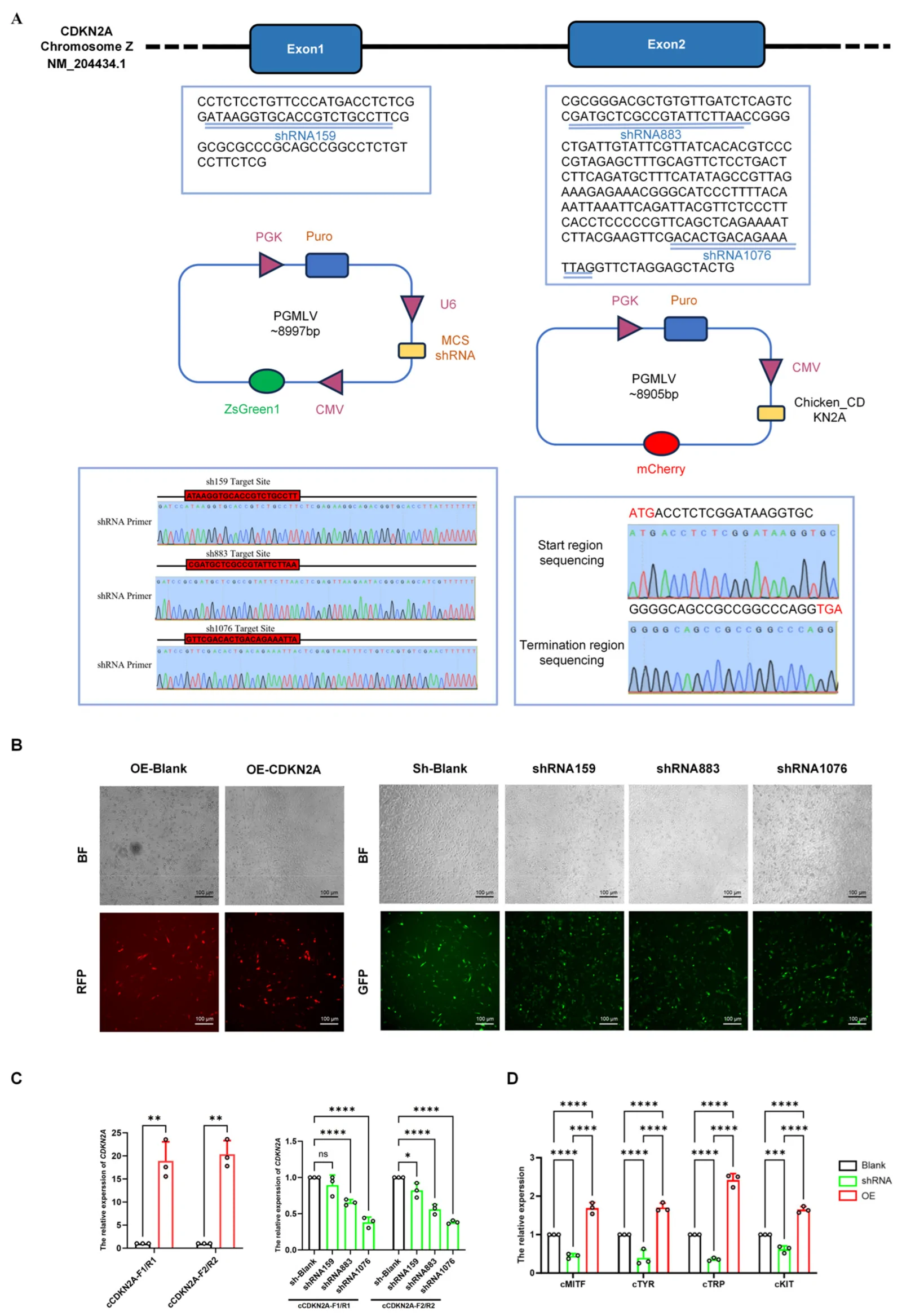
Construction and validation of chicken *CDKN2A* overexpression and knockdown vectors in DF-1 cells: (**A**) Schematic representation and sequencing validation of the *CDKN2A* overexpression vector and three knockdown vectors (shRNA159, shRNA883, and shRNA1076). (**B**) Fluorescence microscopy of DF-1 cells at 48 h post-transfection (red fluorescence indicates the overexpression vector and its negative control, whereas green fluorescence indicates the knockdown vectors and their negative controls). (**C**) Relative expression levels of *CDKN2A* in transfected DF-1 cells determined by qRT-PCR. Statistical significance for the overexpression vector was assessed by unpaired Student’s *t*-test relative to the empty-vector control (NC); statistical significance among the three shRNA constructs and their corresponding NC was assessed by one-way ANOVA followed by Tukey’s post hoc test, with NC as the reference control group. (**D**) Exploratory assessment of transcriptional responses of pigmentation-associated genes following *CDKN2A* modulation in DF-1 cells, as assessed by qRT-PCR. Statistical significance was assessed by one-way ANOVA followed by Tukey’s post hoc test, with the empty-vector-transfected NC group used as the reference control for all comparisons. Data are presented as mean ± SD (*n* = 3 independent experiments); * *p* < 0.05, ** *p* < 0.01, *** *p* < 0.001; **** *p* < 0.0001; ns, not significant.

To investigate the association between *CDKN2A* expression and pigmentation-related transcriptional responses, overexpression and knockdown vectors were constructed. Because DF-1 cells exhibit high transfection efficiency, this cell line was initially used only for validating vector expression and *CDKN2A* manipulation efficiency rather than for evaluating melanogenic functions. First, the full-length coding sequence of *CDKN2A* was amplified using the chicken Z chromosome-linked *CDKN2A* gene (transcript NM_204434.1) as a template. Following removal of the stop codon (TGA), the target fragment was inserted into the multiple cloning site (MCS) downstream of the CMV promoter in the PGMLV-CMV-MCS-mCherry-PGK-Puro vector to generate the overexpression construct, designated PGMLV-CMV-Chicken_*CDKN2A*-mCherry-Puro (Figure 1A).

For gene knockdown, three shRNA target sequences (shRNA159, shRNA883, and shRNA1076) targeting exon 1 and exon 2 of the chicken *CDKN2A* gene were designed. Each target fragment was individually cloned into the MCS downstream of the U6 promoter in the PGMLV-HU6-MCS-CMV-ZsGreen1-PGK-Puro vector. The resulting knockdown constructs were designated Chicken_*CDKN2A*-shRNA159, Chicken_*CDKN2A*-shRNA883, and Chicken_*CDKN2A*-shRNA1076. All constructs were verified by Sanger sequencing, confirming the correct insertion of the full-length *CDKN2A* sequence and corresponding shRNA target sequences into the backbone vectors (Figure 1A).

The functional activities of these constructs were subsequently validated in the DF-1 cell line. At 48 h post-transfection, red fluorescence was detected in cells transfected with the overexpression vector and its control, whereas green fluorescence was observed in cells transfected with the knockdown vectors and their control, indicating successful expression of these constructs in vitro (Figure 1B). The regulatory effects of the constructs on *CDKN2A* transcription were further evaluated via qRT-PCR. The results demonstrated that the overexpression and knockdown vectors significantly increased and decreased *CDKN2A* expression, respectively. Among the three knockdown constructs, shRNA1076 exhibited the highest silencing efficiency and was therefore selected for subsequent transfection experiments in primary melanocytes (Figure 1C).

Furthermore, selected melanogenesis-related transcripts were examined as exploratory markers following *CDKN2A* manipulation in DF-1 cells. The knockdown of *CDKN2A* significantly decreased the expression levels of selected pigmentation-associated genes, including melanogenic enzymes (*TYR*, *TYRP1*), the melanocyte lineage marker *KIT*, and the transcriptional regulator *MITF* (Figure 1D). These observations represented preliminary transcriptional responses following *CDKN2A* manipulation in DF-1 cells rather than direct evidence of melanogenic regulation. Collectively, these results demonstrated that the constructed *CDKN2A* overexpression and knockdown vectors efficiently altered *CDKN2A* expression at the cellular level and provided a basis for further investigation in primary melanocytes.

### 3.2. Isolation and TRP2-Specific Fluorescence Identification of Primary Melanocytes

**Figure 2:**
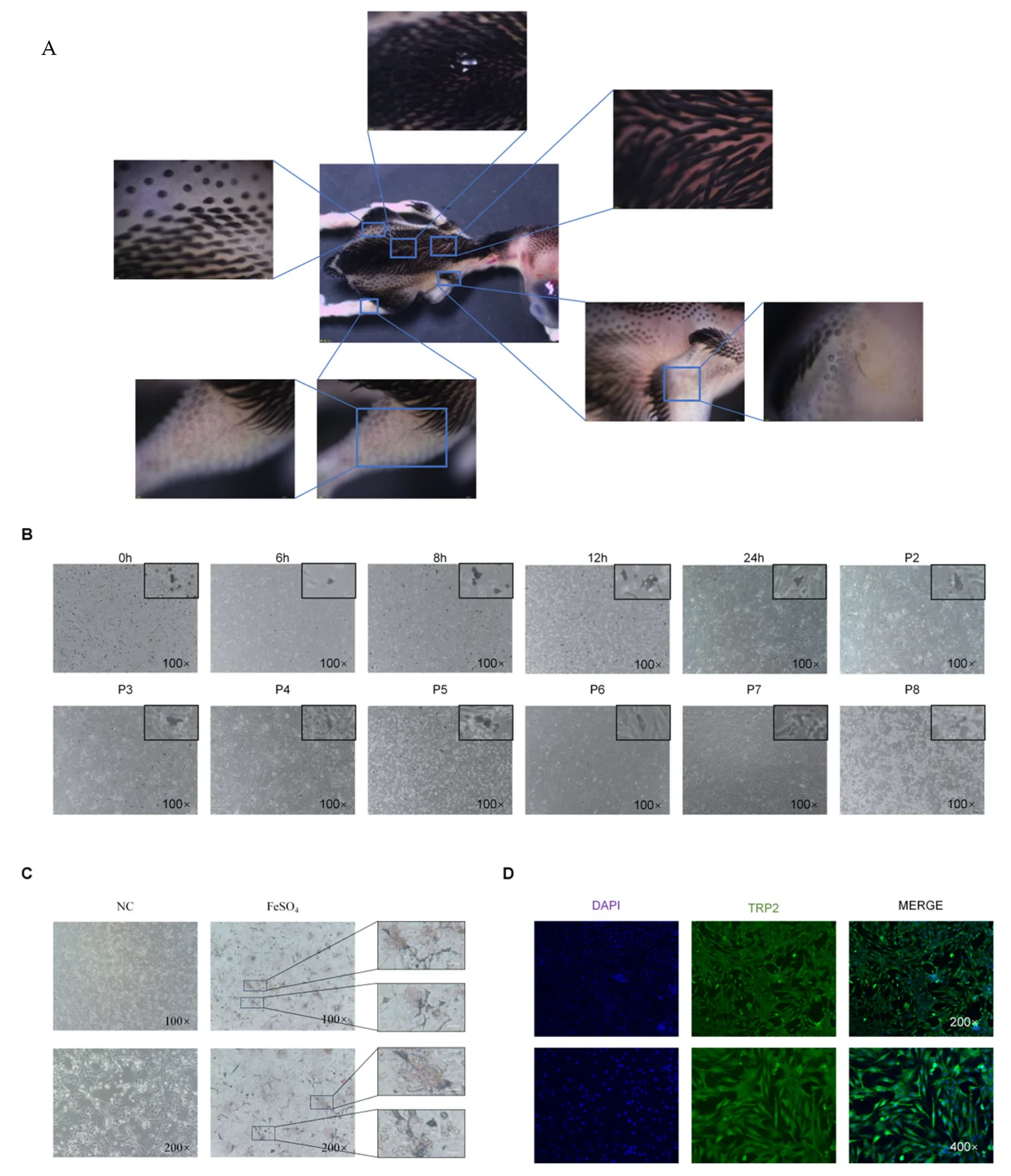
Isolation, culture, and identification of primary melanocytes from Lueyang black-boned chickens: (**A**) Morphological observation of melanin deposition in the feathers (depositing from tip to base) and dorsal skin (exhibiting a spotted distribution) of Lueyang black-boned chickens. (**B**) Dynamic processes of in vitro seeding and adhesion of primary melanocytes (at 0, 6, 8, 12, and 24 h), and micromorphological changes during serial passaging (P2–P8). (**C**) Specific identification of primary melanocytes via ferrous sulfate staining (dark green granules in the cytoplasm indicate sites of melanin deposition and do not colocalize with the red-stained nuclei). (**D**) Expression of the melanocyte-specific marker protein TRP2, as detected by indirect immunofluorescence staining.

To establish an efficient and stable in vitro culture system for chicken primary melanocytes, Lueyang black-boned chickens were utilized as the experimental model. We observed distinct melanin deposition patterns: pigmentation in the feathers progressed from the distal tip toward the base, while dermal pigmentation initially appeared as a distinct spotted pattern on the dorsal skin before extending toward the limbs (Figure 2A). Based on these histological characteristics, melanocytes were specifically isolated from the dorsal skin and peritoneal tissues of Lueyang black-boned chickens [1,15].

Subsequently, the adhesion process and morphological changes of primary melanocytes across successive passages were systematically monitored via phase-contrast microscopy (Figure 2B). At the initial plating stage (0 h), the cells appeared as small, scattered punctate structures. After 6–8 h of culture, the cells gradually adhered to the culture surface and began to spread, exhibiting progressively clearer boundaries. By 12–24 h, the cells were fully attached and extensively spread, displaying enlarged cell bodies, characteristic dendritic processes, and a marked increase in cell density. During serial passaging, cells at passages 2 to 4 (P2–P4) exhibited robust proliferation, favourable growth status, and well-developed, uniform dendritic structures. However, from passage 5 (P5) onward, the cells showed progressive morphological changes consistent with cellular senescence, including reduced dendritic complexity and altered morphology. By passages P7–P8, pronounced degenerative changes were observed, characterized by irregular filamentous structures, cellular shrinkage, and fragmentation, accompanied by extensive apoptosis. These passage-dependent observations indicate that primary melanocytes progressively lose their characteristic morphology after 5–7 passages in vitro, limiting their suitability for long-term culture. Therefore, subsequent experiments should ideally be conducted using early-passage cells (P2–P4).

To further verify the identity and purity of the isolated cells, ferrous sulfate staining and indirect immunofluorescence assays were performed. Following treatment with a ferrous sulfate staining kit, distinct dark green pigment granules were specifically enriched in the cytoplasm without colocalizing with the red-stained nuclei, which is consistent with the characteristics of typical melanin-positive cells [16,17] (Figure 2C). In parallel, fluorescence labelling confirmed that the isolated cell population stably expressed TRP2, a commonly used melanocyte-associated marker protein [18] (Figure 2D).

Collectively, these results demonstrated the successful isolation and identification of primary melanocyte cultures from Lueyang black-boned chickens and characterized their dynamic adhesion processes and passage-dependent morphological changes in vitro. The isolated cells exhibited typical melanocyte-like morphology and expressed the melanocyte-associated marker TRP2, particularly during early passages (P2–P4). These observations established an experimental model suitable for investigating *CDKN2A*-associated transcriptional changes in primary melanocytes.

### 3.3. CDKN2A Expression Modulation Is Associated with Changes in Melanogenesis-Related Marker Expression

**Figure 3:**
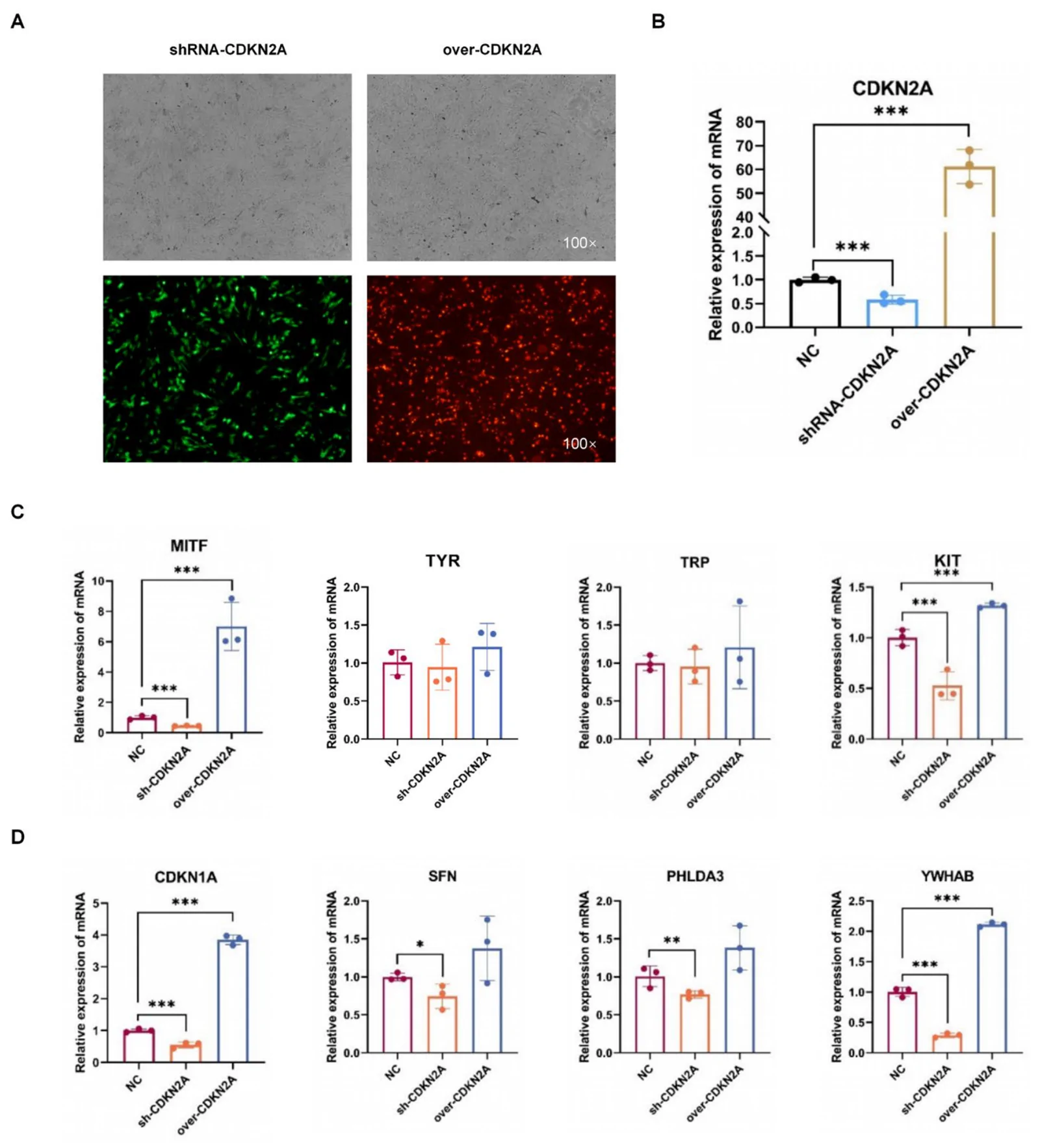
*CDKN2A* expression modulation and associated transcriptional responses in primary melanocytes: (**A**) Transfection of *CDKN2A* knockdown and overexpression vectors into primary melanocytes (green fluorescence indicates the knockdown vector, whereas red fluorescence indicates the overexpression vector). (**B**) Validation of *CDKN2A* knockdown and overexpression efficiency in primary melanocytes by RT-qPCR. Statistical significance was assessed by one-way ANOVA followed by Tukey’s post hoc test across the NC, sh-*CDKN2A*, and over-*CDKN2A* groups, with NC used as the reference control group for calculating relative expression levels. (**C**) Association between *CDKN2A* expression changes and transcriptional levels of selected melanogenesis-related genes (*TYR*, *TYRP1*) and the transcription factor *MITF* in primary melanocytes. *MITF* showed a consistent expression pattern with *CDKN2A*, whereas no significant differences were observed for *TYR* and *TYRP1*. Statistical comparisons among the NC, sh-*CDKN2A*, and over-*CDKN2A* groups were performed by one-way ANOVA followed by Tukey’s post hoc test, with NC as the reference control. (**D**) Transcriptional changes associated with *CDKN2A* knockdown or overexpression in cell cycle- and cellular senescence-associated genes (*CDKN1A*, *YWHAB*, *SFN*, and *PHLDA3*). Statistical significance was determined by one-way ANOVA followed by Tukey’s post hoc test relative to the NC group. Data are presented as mean ± SD (n = 3 independent experiments); * *p* < 0.05, ** *p* < 0.01, *** *p* < 0.001.

To further investigate the transcriptional responses associated with *CDKN2A* expression in primary melanocytes, the validated knockdown vector (shRNA1076) and overexpression vector (OE-*CDKN2A*) were transfected into cultured primary melanocytes from theLueyang black-boned chicken (Figure 3A). RT-qPCR analysis confirmed that *CDKN2A* expression was significantly reduced or increased following knockdown or overexpression, respectively (Figure 3B).

Following *CDKN2A* modulation, the expression patterns of selected melanogenesis-related genes were examined by RT-qPCR. In primary melanocytes, *MITF* expression showed a similar directional trend to *CDKN2A* expression, with *CDKN2A* knockdown decreasing *MITF* transcript levels, whereas *CDKN2A* overexpression increased *MITF* expression. However, the expression changes of downstream melanogenic enzymes, including *TYR* and *TYRP1*, were relatively limited in primary melanocytes and did not reach statistical significance by RT-qPCR, suggesting that these genes may represent modest transcriptional responses following *CDKN2A* modulation (Figure 3C). In addition, the expression of cell cycle- and senescence-associated genes was evaluated. *CDKN2A* overexpression or knockdown resulted in corresponding changes in several senescence-associated genes, including *CDKN1A*, *YWHAB*, *SFN*, and *PHLDA3* (Figure 3D).

Overall, these results showed that *CDKN2A* expression changes were associated with alterations in *MITF* expression and cell-state-related transcriptional responses in primary melanocytes, whereas the effects on downstream melanogenic enzymes were relatively modest.

### 3.4. Transcriptomic Changes Associated with CDKN2A Expression Fluctuations and Differential Gene Enrichment Analysis

**Figure 4:**
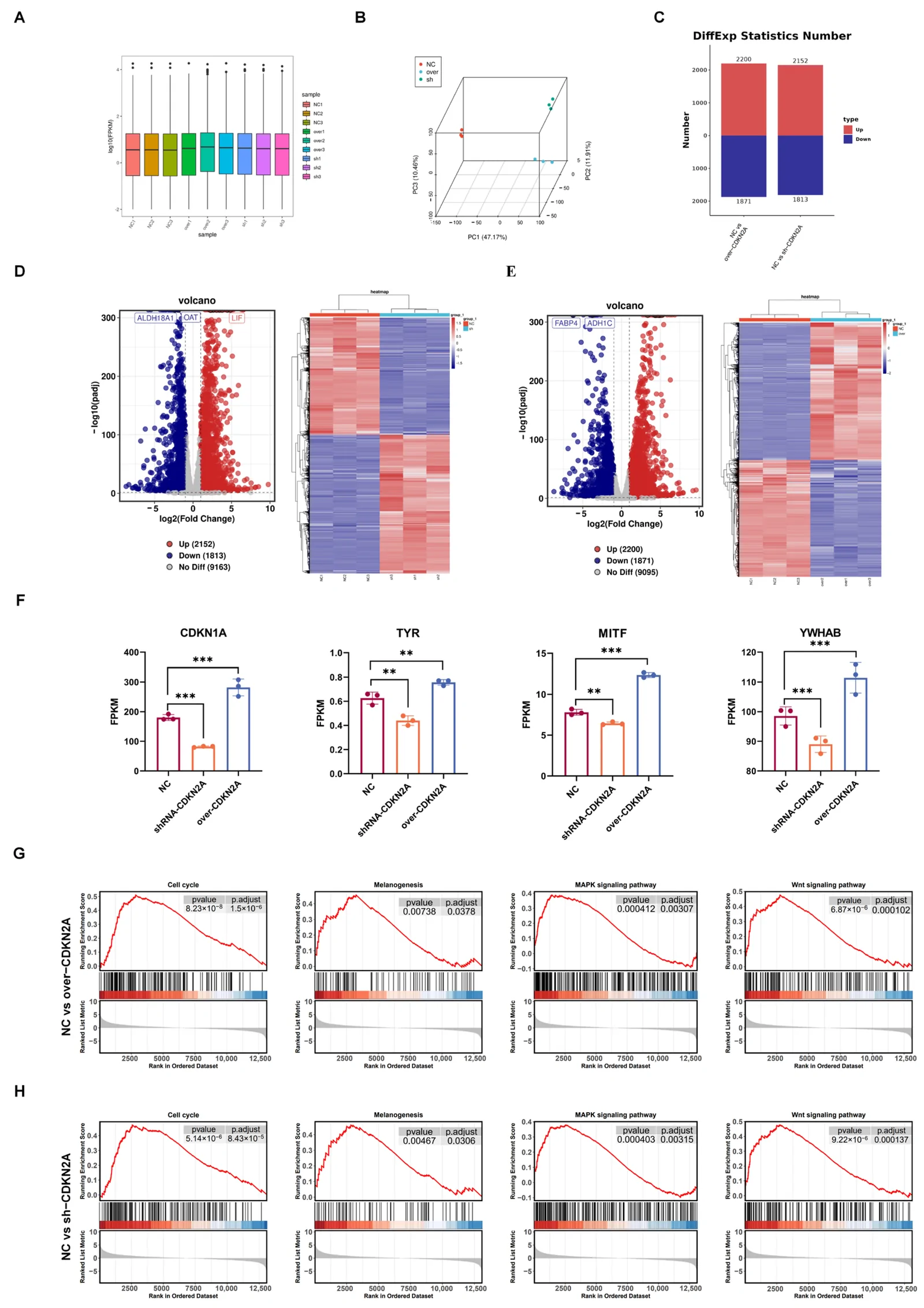
Transcriptomic analysis associated with *CDKN2A* knockdown and overexpression: (**A**) Box plots showing normalized expression distributions across different samples. (**B**) Three-dimensional principal component analysis (3D PCA) plot, illustrating the spatial clustering of biological replicates among groups. (**C**) Statistical summary of the number of differentially expressed genes (DEGs) between the control and treatment groups. (**D**) Volcano plot and hierarchical clustering heatmap of DEGs between the NC and sh-*CDKN2A* groups. (**E**) Volcano plot and hierarchical clustering heatmap of DEGs between the NC and over-*CDKN2A* groups. (**F**) Expression patterns of selected melanogenesis-associated (*MITF*, *TYR*) and cell cycle-associated (*CDKN1A*, *YWHAB*) genes following *CDKN2A* knockdown or overexpression, relative to the NC group. Statistical significance for panel (**F**) was determined by DESeq2-based differential expression analysis with FDR correction (adjusted *p* < 0.05), consistent with the criteria used for overall DEG identification (panels (**C**–**E**)), rather than by *t*-test or ANOVA. ** indicates adjusted *p* < 0.01 and *** indicates adjusted *p* < 0.001. (**G**,**H**) Gene set enrichment analysis (GSEA) profiles showing significant enrichment of gene sets associated with the cell cycle, melanogenesis, MAPK, and Wnt signalling pathways (GSEA-derived enrichment significance, adjusted *p* < 0.05, distinct from the DEG-level statistics in panels (**C**–**F)**).

To characterize the transcriptional alterations associated with *CDKN2A* modulation in melanocytes, RNA-seq was performed using primary melanocytes transfected with *CDKN2A* overexpression or knockdown vectors (Figure 4). The expression distribution and principal component analysis (PCA) demonstrated clear separation among the NC, over-*CDKN2A*, and sh-*CDKN2A* groups, indicating distinct transcriptional profiles among experimental conditions (Figure 4A,B).

Compared with the NC group, 2200 and 2152 differentially expressed genes (DEGs) were identified in the over-*CDKN2A* and sh-*CDKN2A* groups, respectively (Figure 4C). Volcano plots and hierarchical clustering analysis further demonstrated distinct transcriptional patterns associated with *CDKN2A* expression changes (Figure 4D,E). Functional enrichment analysis of the identified DEGs revealed significantly enriched Gene Ontology (GO) terms and Kyoto Encyclopedia of Genes and Genomes (KEGG) pathways in both the sh-*CDKN2A* and over-*CDKN2A* groups (Figure S1).

Among the identified DEGs, representative genes associated with melanocyte function and cell-state regulation, including *MITF*, *TYR*, *CDKN1A*, and *YWHAB*, were selected for further examination based on their reported biological relevance to melanocyte biology. Their expression patterns showed changes consistent with *CDKN2A* modulation (Figure 4F). However, although *TYR* showed differential expression patterns in the RNA-seq analysis, these changes should be interpreted cautiously because the magnitude of alteration was relatively modest and was not statistically confirmed by RT-qPCR validation.

GSEA revealed that gene sets related to cell cycle regulation, melanogenesis, MAPK signalling, and Wnt signalling were enriched in the comparisons between *CDKN2A*-modulated groups and controls (adjusted *p* < 0.05) (Figure 4G,H).

## 4. Discussion

This study investigated the expression characteristics and potential biological relevance of the Z chromosome-linked *CDKN2A* gene in primary melanocytes of theLueyang black-boned chicken. By integrating qRT-PCR, RNA-seq, and primary melanocyte analyses, we found that *CDKN2A* expression was associated with changes in melanogenesis-related transcriptional programs, particularly *MITF* expression. These findings provided transcriptional evidence suggesting a potential relationship between *CDKN2A* expression and melanocyte state regulation. However, the present study primarily identified *CDKN2A*-associated transcriptional alterations rather than demonstrating direct regulation of pigmentation. Therefore, the observed changes should be interpreted as molecular associations that require further functional validation.

*MITF*, a key transcription factor involved in melanogenesis, regulates the expression of downstream melanogenic enzymes, including *TYR* [19]. In the present study, differences were observed between RNA-seq and RT-qPCR measurements for *TYR* and *TYRP1* expression. Although transcriptomic analysis indicated altered expression patterns of these genes, these changes were not statistically confirmed by targeted RT-qPCR analysis. Therefore, *TYR* expression changes identified by RNA-seq may represent subtle transcriptional responses rather than strong downstream effects of *CDKN2A*. Such differences highlight the complexity of interpreting transcriptomic data from primary melanocyte cultures, in which technical variation and cellular heterogeneity may influence gene expression measurements.

From a technical perspective, differences in normalization methods, sequencing depth, and detection sensitivity between RNA-seq and RT-qPCR may contribute to variations in quantitative results [20]. From a biological perspective, primary melanocytes contain heterogeneous populations with distinct transcriptional states, which may introduce variability in gene expression measurements [21]. In addition, transcriptional buffering, post-transcriptional regulation, and cellular plasticity may affect the relationship between transcript abundance and downstream biological response [22,23,24]. Therefore, RNA-seq-derived DEGs should be considered candidate transcriptional responses, and additional validation of representative transcriptome-wide changes will be necessary to confirm their biological relevance.

Previous studies have established that *MITF* functional activity is regulated not only at the transcriptional level but also through intracellular signalling pathways and post-translational mechanisms. For instance, MAPK signalling regulates *MITF* activity through phosphorylation, while transcriptional co-regulators such as p300/CBP contribute to *MITF*-mediated transcriptional regulation [25,26,27]. These findings suggest that changes in *MITF* transcript levels may not always result in proportional alterations of downstream melanogenic enzymes, highlighting the complexity of melanogenesis regulation. Therefore, the association between *CDKN2A* and *MITF* expression observed in this study may reflect changes in melanocyte cellular states rather than a direct effect on melanin synthesis, particularly considering that downstream melanogenic genes such as *TYR* exhibited relatively modest transcriptional alterations.

GSEA suggested that *CDKN2A*-associated transcriptional changes were enriched in melanogenesis-, MAPK-, and Wnt-related gene sets. Previous studies have suggested that MAPK signalling participates in melanocyte functional maintenance, whereas Wnt signalling plays important roles in melanocyte development and progenitor cell regulation [28,29,30]. These results indicated that *CDKN2A* expression changes were associated with transcriptional alterations in pathways related to melanocyte biology. Nevertheless, enrichment analysis reflects coordinated changes in gene expression profiles rather than direct pathway activation. Therefore, these findings should be considered potential pathway associations requiring further experimental confirmation.

Protein-level validation and pathway-specific functional assays will be necessary to determine whether these transcriptional changes are translated into corresponding molecular and cellular effects. Such studies will help clarify whether *CDKN2A*-associated transcriptional responses contribute directly to melanocyte differentiation, maintenance, or pigmentation regulation.

In addition to pigmentation-associated genes, *CDKN2A* expression changes were associated with transcriptional alterations in several cell cycle- and cell-state-related genes, including *CDKN1A*, *SFN* [31], *PHLDA3* [32], *YWHAB* [33], and *KIT*. *CDKN2A*, particularly the p16INK4a protein, is a well-established regulator of cell cycle arrest and cellular senescence. Previous studies have suggested that alterations in *CDKN2A* expression may influence cellular proliferation and differentiation states in different biological systems. In the present study, *CDKN2A* modulation was associated with changes in senescence-related gene expression in primary melanocytes. However, the current data did not distinguish whether these changes represented physiological differentiation-associated processes or senescence-related responses. Because cellular senescence may influence cellular morphology, metabolism, and pigmentation-associated features, further studies incorporating senescence-specific assays, such as senescence-associated β-galactosidase analysis, will be necessary to clarify this relationship.

Regarding breed-specific differences, previous studies have demonstrated that regulatory variants in the Z-chromosome region of SLB chickens can affect p16/ARF expression and melanocyte cell cycle regulation [34,35,36]. In contrast, the pigmentation traits of theLueyang black-boned chicken have been primarily associated with classical pigmentation-related genes such as *ASIP*, *TYR*, and *KIT* [37]. These differences suggest that the biological contribution of *CDKN2A*-associated transcriptional changes may vary among genetic backgrounds. Therefore, further investigations involving additional chicken populations and in vivo models will be required to determine the broader relevance of *CDKN2A* in avian pigmentation.

From a broader biological perspective, *CDKN2A* may represent a potential link between cell cycle regulation and pigmentation-associated cellular states. Previous studies have shown that α-MSH signalling can induce p16-mediated cell-cycle responses under ultraviolet stress, while α-MSH itself promotes melanogenesis [38,39]. These observations suggest a possible interaction between cell cycle regulation and pigment synthesis during stress-induced pigmentation responses. However, whether this regulatory relationship is conserved in avian melanocytes remains unclear, and the present study provides only transcriptional evidence supporting a possible association between *CDKN2A*-related cellular states and pigmentation-associated programs.

This study had several limitations. First, although changes in melanogenesis-related gene expression were observed, direct functional measurements of pigmentation, including melanin content quantification and tyrosinase activity assays, were not performed. Therefore, the present findings should be interpreted as transcriptional evidence associated with *CDKN2A* expression rather than direct evidence of altered melanin production. Second, although representative genes identified from RNA-seq analysis were examined by RT-qPCR, comprehensive validation of additional differentially expressed genes was not performed. Therefore, further validation of transcriptome-wide candidate genes will be necessary to strengthen the interpretation of RNA-seq results and confirm the reproducibility of *CDKN2A*-associated transcriptional changes. Third, protein-level validation was not performed in this study. Since mRNA expression does not always correspond directly to protein abundance or functional activity due to post-transcriptional regulation, future studies incorporating Western blotting, immunofluorescence, or other protein-based approaches will be required to determine whether transcriptional alterations are reflected at the protein level. However, technical limitations associated with primary chicken melanocyte cultures, including limited availability of validated *Gallus gallus*-specific antibodies and challenges in obtaining sufficient biological material for reliable quantitative analyses, restricted reliable protein-level validation in the current study. Furthermore, the transient *CDKN2A* overexpression system resulted in a marked increase in *CDKN2A* transcript abundance. Although no obvious cell loss or severe morphological abnormalities were observed during the experimental period, potential effects associated with supraphysiological *CDKN2A* expression cannot be completely excluded. Finally, the primary melanocyte culture system reduced potential interference from tissue-level interactions but could not fully reproduce the complexity of in vivo pigmentation regulation. Although the isolated cells were characterized by morphology, melanin staining, and TRP2 immunofluorescence, the purity of the primary melanocyte population was not quantitatively assessed using approaches such as flow cytometry. Therefore, the potential contribution of other skin-derived cell populations cannot be completely excluded. However, the enrichment of TRP2-positive cells and the consistency of melanocyte-associated characteristics support the suitability of this model for investigating *CDKN2A*-associated cellular responses in chicken melanocytes. Further validation using more comprehensive cell characterization, tissue-level analyses, and animal models will be required to determine the physiological relevance of these findings.

In summary, this study provided expression-level evidence indicating that *CDKN2A* was associated with pigmentation-related transcriptional programs and broader cell-state regulatory networks in Lueyang black-boned chicken melanocytes. *CDKN2A*-associated changes involved melanogenesis-related genes, cell cycle-associated genes, and pathway-related transcriptional alterations. Importantly, these findings represent transcriptional associations rather than definitive evidence of direct *CDKN2A*-mediated regulation of pigmentation. However, the direct molecular mechanisms underlying these associations and their functional consequences in pigment formation remain to be further determined.

## 5. Conclusions

In conclusion, this study established an in vitro primary melanocyte culture system derived from theLueyang black-boned chicken and revealed an association between *CDKN2A* expression and melanogenesis-related transcriptional programs. *CDKN2A* expression showed a consistent relationship with *MITF* expression and was associated with transcriptional changes related to melanocyte cell-state regulation. RNA-seq enrichment analysis further identified associations between *CDKN2A* expression changes and MAPK- and Wnt-related gene sets. However, these findings represent potential pathway associations rather than confirmed pathway regulation or direct functional effects on pigmentation. This study provided transcriptomic evidence for understanding pigmentation-associated molecular networks in poultry. Future studies integrating transcriptomic, proteomic, and functional approaches will be necessary to clarify the precise biological role of *CDKN2A* in melanocyte development, cellular state regulation, and pigment formation.

## Figures and Tables

**Table 1:** Primer sequences used for RT-qPCR in this study.

Gene/Target	Forward/Reverse Primer Sequence (5′ to 3′)	Product Size (bp)
c*CDKN2A*-1	F:CCCGTAGAGCTTTGCAGTTC R:ACATGCAGGAACGGATCTTC	228
c*CDKN2A*-2	F:GACATTGTCGGGGGACTCTA R:TGGTGTGAACACTGGGAAGA	155
c*MITF*	F:TGTCCCTTGTTCCATCC R:ACATTTCCCAGGTTGTCAC	204
c*TYR*	F:TCAAGGACCCCAAGACTCCC R:CGGAAGCTGTAATTGGCCATT	106
*cTYR* *P* *1*	F:GCTAAGAAGGTATTCGGCT R:AGTGAGAAGAGGCTGATGC	376
c*KIT*	F:CATCGGTGCCATCCTTTGAG R:CTTGTCACCATTGCTGGATGG	110
*β-actin*	F:CAGCCATCTTTCTTGGGTAT R:CTGTGATCTCCTTCTGCATCC	169

F: forward primer; R: reverse primer. The “c” prefix indicates chicken (*Gallus gallus*) genes. *β-actin* was used as the internal reference gene.

## Data Availability

The RNA-seq datasets generated in this study have been submitted to the NCBI Sequence Read Archive (SRA) database and will be publicly available upon release. The accession number will be provided after the data become available.

## Supplementary Materials

The following supporting information can be downloaded at https://www.mdpi.com/article/10.3390/ani16162483/s1, Figure S1: GO and KEGG enrichment analysis of differentially expressed genes. (A) GO terms and KEGG pathways enriched in the NC vs. sh-*CDKN2A* comparison group. (B) GO terms and KEGG pathways enriched in the NC vs. over-*CDKN2A* comparison group.

## Abbreviations

The following abbreviations are used in this manuscript:

*CDKN2A*	Cyclin-dependent kinase inhibitor 2A
RT-qPCR	Reverse transcription quantitative polymerase chain reaction
RNA-seq	RNA sequencing
PCA	Principal component analysis
FPKM	Fragments per kilobase of transcript per million mapped reads
GSEA	Gene set enrichment analysis
DEGs	Differentially expressed genes
GO	Gene Ontology
KEGG	Kyoto Encyclopedia of Genes and Genomes
MAPK	Mitogen-activated protein kinase
*MITF*	Microphthalmia-associated transcription factor
*TYR*	*Tyr*osinase
*TYRP1*	*Try*osinase-related protein 1
SLB	Sex-linked barring
IACUC	Institutional Animal Care and Use Committee
SRA	Sequence Read Archive
α-MSH	α-melanocyte-stimulating hormone
