# Supplementary material for: Transcriptomic Analysis Identifies CDKN2A-Associated Transcriptional Alterations in Primary Melanocytes of Lueyang Black-Boned Chicken
Source: Animals (Basel). 2026 Aug 10;16(16):2483. doi: 10.3390/ani16162483 (PMC13508894; doi:10.3390/ani16162483)
Supplement: Supplementary file 1 [file animals-16-02483-s001.zip › animals-4451614-supplementary.pdf]

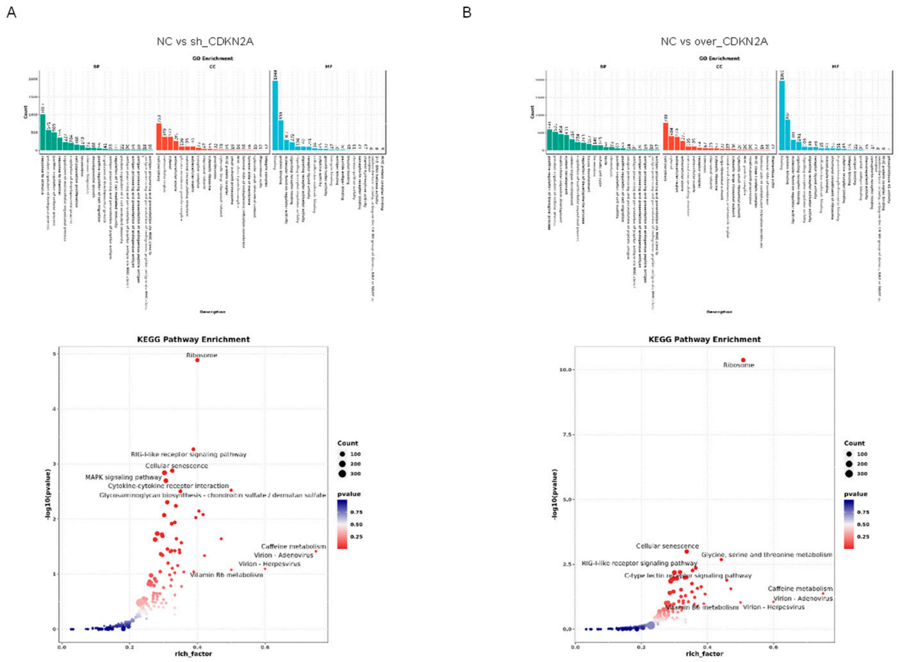

Figure S1. GO and KEGG enrichment analysis of differentially expressed genes. (A) GO terms and KEGG pathways enriched in the NC vs. sh-CDKN2A comparison group.(B) GO terms and KEGG pathways enriched in the NC vs. over-CDKN2A comparison group.
